# Supplementary material for: Surface Microbiome Profiling of Dental Elevators Using Third‐Generation Sequencing: Implications for Infection Control in Dental Practice
Source: Microbiologyopen. 2025 Dec 8;14(6):e70178. doi: 10.1002/mbo3.70178 (PMC12685768; doi:10.1002/mbo3.70178)
Supplement: Supplementary file 1 — customer_backup. [file MBO3-14-e70178-s001.zip › customer_backup/customer_backup/taxa_summary/krona/groups/treat/M06.Krona.html]

Javascript must be enabled to view this page.

members
magnitude
magnitudeUnassigned

M06.krona

53197

53190

1

0

0

0

0

0

0

0

0

0

0

0

0

0

0

0

1

1

1

1

0

0

0

0

0

0

0

0

1

0

0

0

0

0

0

0

0

0

0

0

0

0

0

2

1

0

0

0

0

0

0

0

0

0

0

0

0

0

0

0

0

0

1

0

0

0

0

0

0

0

0

0

0

0

0

0

0

0

0

0

0

0

0

0

0

0

0

0

0

1

1

1

0

0

1

0

0

0

0

0

0

0

1

1

1

1

0

0

0

0

0

0

0

0

0

0

0

0

67

3

3

0

0

0

3

1

1

0

2

2

0

0

0

0

0

0

0

0

0

0

0

0

0

0

0

0

0

0

0

0

0

0

0

0

0

0

0

0

0

0

0

0

0

0

0

0

0

0

0

0

0

0

0

0

0

0

0

0

0

0

0

0

0

0

0

64

64

0

0

0

0

0

0

0

0

0

0

0

0

0

0

0

0

0

0

0

0

0

0

0

0

64

0

0

0

0

0

64

64

0

0

0

0

0

0

0

0

0

0

0

0

0

0

0

0

0

0

0

0

0

0

0

0

0

0

0

0

0

0

0

0

0

0

0

0

0

0

0

0

0

0

0

0

0

0

0

0

0

0

0

0

0

0

0

0

0

0

0

0

0

0

0

0

0

0

0

0

0

0

0

0

0

0

0

0

0

0

0

0

0

0

0

0

0

0

0

0

0

0

0

0

0

0

0

0

0

0

0

0

0

0

0

0

0

0

0

13

0

0

0

0

0

0

0

0

0

0

0

0

0

0

0

0

0

0

0

0

0

0

0

0

13

1

1

0

0

0

0

1

0

1

0

0

0

0

0

0

0

0

0

0

0

0

0

0

0

12

1

0

0

0

0

1

1

0

10

0

0

10

10

0

0

0

0

0

0

0

0

0

0

0

1

1

0

1

0

0

0

0

0

0

0

0

0

0

0

0

0

0

0

0

0

0

1490

128

0

0

0

0

0

0

0

0

0

0

0

0

0

0

0

0

0

0

0

0

0

0

0

0

1

1

1

0

1

0

0

0

0

0

0

0

0

0

0

0

0

0

0

0

0

0

0

0

0

1

1

0

0

0

0

1

0

0

0

1

0

0

0

0

0

0

0

8

8

0

0

0

0

0

0

0

0

0

0

0

0

0

0

1

1

0

0

0

0

0

0

0

0

0

0

0

0

0

0

0

0

1

0

0

1

0

0

0

0

6

0

0

0

0

0

0

0

0

0

0

0

0

3

0

0

0

0

0

0

0

1

0

0

0

2

0

0

0

0

0

0

0

0

0

0

0

0

0

0

0

0

0

0

0

56

0

0

0

0

0

0

0

0

0

0

0

0

0

0

0

0

0

0

0

0

0

1

0

0

0

1

0

1

0

0

0

0

0

0

0

0

0

0

0

0

0

0

0

0

0

0

0

0

0

0

0

40

0

0

0

0

0

0

0

8

8

0

0

0

0

19

19

0

0

0

0

0

0

0

8

0

1

7

0

0

0

0

0

0

5

3

0

0

0

0

0

0

0

0

0

0

2

0

0

0

0

0

0

0

0

0

0

0

0

0

0

0

0

0

0

0

0

0

0

0

0

0

4

1

0

0

1

0

0

0

0

0

3

0

2

1

0

0

0

0

0

0

0

0

3

0

0

0

0

0

2

2

0

0

0

1

1

0

0

0

0

0

0

0

0

0

0

0

0

0

8

2

2

0

0

0

0

0

0

0

0

0

0

1

0

0

0

0

0

0

0

0

0

1

0

0

0

0

0

0

1

1

0

0

0

0

0

0

0

0

0

4

4

1

1

0

0

1

1

1

0

0

0

0

0

1

1

0

0

1

0

0

0

0

0

0

0

0

0

0

0

0

0

0

0

0

0

0

0

0

0

0

0

0

0

0

0

0

0

0

0

0

0

0

0

0

0

0

0

0

0

0

0

0

0

0

0

0

0

0

0

0

0

0

0

0

0

0

0

0

0

0

0

0

0

0

59

59

0

0

0

0

0

59

59

0

0

0

0

0

0

0

0

0

0

0

0

0

0

0

0

0

0

0

0

0

0

0

0

0

0

0

0

0

1

0

0

0

0

0

0

0

0

0

0

0

0

1

1

1

0

0

0

0

0

0

0

0

0

0

0

0

0

0

0

0

0

0

0

0

0

0

0

0

0

0

0

0

0

0

0

0

0

0

0

0

0

0

0

0

0

0

0

0

0

0

0

0

0

0

0

0

0

0

0

0

0

0

0

0

0

0

0

0

1362

0

0

0

0

0

0

0

0

0

0

0

0

0

0

0

0

0

0

0

990

0

0

0

0

0

0

0

0

0

0

0

0

6

0

0

0

0

0

0

0

0

0

6

6

0

0

0

0

0

0

0

0

0

0

0

408

0

0

0

0

407

407

0

0

0

0

0

1

0

0

0

0

0

1

0

0

0

0

0

0

0

0

0

0

0

0

0

0

0

0

0

0

0

0

0

0

0

0

0

0

0

0

0

18

0

0

6

6

0

0

0

0

0

0

0

0

0

0

12

12

26

19

19

0

0

6

0

6

1

0

1

0

0

0

0

532

0

0

0

0

0

0

0

0

0

0

0

0

0

0

0

0

0

0

0

0

0

241

0

241

0

0

0

0

0

41

0

41

0

0

0

42

7

35

208

208

0

0

0

0

0

0

0

0

1

0

0

0

0

0

0

0

0

1

1

0

1

0

0

0

0

0

0

0

0

0

0

0

0

0

0

0

199

199

199

130

69

0

0

0

0

1

1

1

1

0

0

0

0

0

0

0

0

0

0

0

38

0

0

0

14

0

0

0

0

0

0

0

14

14

0

0

0

0

0

0

0

0

0

0

0

0

0

0

0

0

0

0

0

0

0

0

0

0

0

0

24

24

0

0

23

1

0

0

0

0

0

0

0

0

0

0

0

0

0

0

0

0

0

0

0

0

0

0

0

0

0

0

0

0

0

0

0

0

0

0

0

0

0

0

0

0

0

0

0

0

0

0

0

0

0

0

0

0

0

0

0

0

0

0

114

1

0

0

1

1

0

0

0

0

0

0

0

0

0

0

0

0

9

0

0

4

4

4

4

1

1

0

0

0

0

3

3

3

0

0

0

2

2

2

99

0

0

0

0

5

5

0

0

31

0

8

23

0

63

63

0

0

0

0

0

0

0

0

0

0

0

0

1

1

1

1

0

0

0

0

0

0

0

0

0

0

0

0

0

0

0

0

0

0

6

1

0

0

0

0

0

0

0

1

1

0

0

0

5

3

3

0

0

0

0

0

0

0

0

0

0

0

0

2

0

0

1

0

0

0

1

0

0

0

0

0

0

0

0

0

0

0

0

0

0

0

0

0

0

0

0

0

0

0

0

0

0

0

0

9

9

0

0

0

0

0

0

0

0

0

0

0

0

9

3

0

6

0

0

2

2

2

2

0

0

0

0

1

0

0

0

0

0

0

1

1

1

0

0

0

0

0

0

0

0

0

0

0

0

5

5

5

0

0

0

5

1

1

0

0

0

0

0

0

4

0

4

0

0

0

0

0

0

0

0

0

0

0

0

0

0

0

0

0

0

0

0

0

0

0

0

0

0

0

0

0

0

0

0

0

0

0

0

0

0

0

0

0

0

0

0

0

0

0

0

0

0

0

0

0

0

0

0

0

0

0

0

0

0

0

0

0

0

0

0

0

0

0

0

0

0

0

0

0

0

0

0

0

0

0

0

0

0

0

0

0

0

0

0

0

0

0

0

0

0

0

0

0

0

0

0

0

0

0

0

0

9396

0

0

0

0

0

9396

1

0

0

0

1

0

0

0

0

0

0

0

0

0

0

0

0

0

0

0

0

0

0

0

0

0

0

0

0

0

0

0

0

0

0

1

0

0

1

0

0

0

0

0

0

0

0

0

0

0

0

0

0

0

0

0

0

0

0

0

0

0

0

0

0

0

0

755

0

0

0

744

0

0

0

0

0

0

0

0

0

0

0

0

0

0

0

0

0

0

0

0

0

744

2

50

636

56

0

0

0

0

0

0

0

0

0

0

0

0

0

0

0

0

0

0

0

6

6

0

6

0

0

0

0

0

5

0

0

0

0

5

5

0

0

8475

12

0

0

12

12

5

5

5

7

7

7

75

0

0

2

2

28

28

45

45

34

0

0

0

0

0

33

0

2

0

31

0

0

0

1

0

0

0

0

0

0

0

1

0

0

1005

1005

1005

0

0

0

0

0

0

0

0

0

0

0

0

0

0

0

0

0

9

9

0

1

1

0

0

0

0

0

0

0

0

1

4

2

0

0

0

652

0

0

651

596

55

1

1

0

0

0

0

0

4808

0

0

0

0

0

0

519

56

0

16

444

3

2

2

0

0

0

0

2150

18

451

85

185

6

0

177

17

12

4

80

10

60

39

0

0

1006

2137

0

52

56

209

408

208

0

0

0

0

351

62

25

0

190

576

0

0

0

0

0

0

1631

1631

0

610

0

990

0

31

0

0

0

237

237

237

163

0

0

0

0

0

0

0

0

0

0

0

0

163

0

0

0

0

0

0

163

24

139

0

0

0

2

0

0

0

0

0

0

0

0

0

0

0

0

0

0

0

0

0

0

0

0

0

0

0

0

0

0

0

0

0

0

0

0

0

0

0

0

0

0

2

1

1

1

1

0

0

0

0

0

0

0

0

0

0

0

0

0

0

0

0

0

0

0

0

0

0

0

0

0

0

0

0

0

0

0

0

0

0

0

0

0

0

0

0

0

0

0

0

0

0

0

0

0

0

0

0

0

0

0

0

0

0

0

0

0

0

0

0

0

0

0

0

0

0

0

0

0

0

0

0

0

0

0

0

0

0

0

0

0

0

0

0

0

0

0

0

0

0

0

0

0

0

0

0

0

0

0

0

0

0

0

0

0

0

0

0

0

0

0

0

0

0

0

0

0

0

0

0

0

0

0

0

0

0

0

0

0

0

4264

3145

3145

0

0

0

3095

262

228

0

0

34

2833

276

2296

251

10

50

12

12

1

0

0

0

1

2

2

0

0

8

8

0

20

20

0

0

0

3

0

0

3

0

0

0

0

4

4

1

1

1

1

1

12

6

6

0

0

0

0

6

6

0

0

0

0

0

0

0

0

0

0

0

0

2

0

0

0

0

0

0

2

2

2

0

0

0

0

0

0

0

0

0

0

0

0

0

0

0

0

0

0

0

0

0

0

0

0

0

0

0

0

0

0

0

0

4

0

0

0

0

0

0

0

0

0

0

0

0

0

0

0

0

2

0

0

0

0

0

0

2

1

1

2

0

0

0

0

2

2

9

6

0

0

0

4

0

0

0

0

0

0

0

0

0

0

0

0

0

0

0

0

0

0

0

0

0

4

4

2

0

0

0

0

2

2

0

0

0

0

3

2

2

1

0

1

1

0

0

0

0

1

1

1097

0

0

0

0

0

0

0

0

0

0

0

0

0

0

0

0

0

0

0

0

0

0

0

0

0

0

0

0

0

0

0

0

0

0

0

0

0

0

0

0

0

0

0

0

0

0

0

0

0

0

0

0

0

0

0

0

0

0

0

0

0

0

17

17

17

0

17

0

0

0

0

0

0

0

0

0

0

0

0

0

0

0

0

0

0

0

0

667

667

580

0

580

68

0

7

11

0

0

1

46

3

19

19

0

0

0

0

0

0

0

0

0

0

0

0

0

0

0

0

0

0

0

0

0

0

0

0

0

0

0

0

0

0

0

0

0

0

0

0

0

0

0

0

0

0

0

0

179

179

4

4

0

0

175

1

19

1

0

6

55

0

0

0

11

15

0

67

0

0

0

0

0

0

0

0

0

0

0

0

0

0

0

0

0

0

0

168

167

0

0

0

0

123

123

20

20

4

4

0

0

0

0

0

0

20

0

20

0

0

0

1

0

0

0

0

0

0

1

0

0

0

0

0

0

1

0

0

0

0

0

0

0

0

0

0

0

0

0

0

0

0

0

0

0

0

0

0

0

4

4

0

0

0

0

4

1

1

2

0

0

0

0

0

0

0

0

0

0

0

0

0

0

0

0

0

0

0

0

0

62

53

1

1

48

48

1

0

1

0

0

1

1

0

2

0

1

0

1

0

0

0

0

0

0

0

0

0

0

0

0

0

0

0

0

0

0

0

0

0

0

0

0

0

0

0

1

1

1

0

0

0

0

7

0

0

0

0

0

0

1

0

1

0

0

0

0

0

0

0

0

0

0

0

0

0

0

1

1

5

0

0

5

0

0

0

0

0

0

0

0

0

0

0

1

1

1

0

0

0

0

0

0

0

0

0

0

1597

1597

1597

1597

0

0

1597

0

0

0

98

0

330

36

291

1

310

322

0

1

0

0

0

0

0

0

208

0

0

0

0

0

0

0

0

0

0

0

0

0

0

0

0

0

16

16

16

16

16

16

9865

9865

9865

9619

10

10

0

0

0

0

9609

8356

1253

246

246

32

38

57

13

10

17

23

56

0

3937

3937

3937

0

0

0

0

0

0

0

3937

3937

192

3728

17

0

0

0

0

0

0

0

0

0

1

0

0

0

0

0

1

1

0

0

0

1

1

1

0

0

0

0

0

0

0

0

0

0

0

0

0

1

1

1

1

1

1

0

0

0

3

1

0

0

0

0

1

1

1

1

0

0

0

0

0

0

0

0

0

0

0

0

0

0

0

0

1

0

0

0

0

0

0

0

0

0

0

0

0

0

0

1

1

0

0

1

0

0

1

0

0

0

0

0

0

0

0

0

0

0

0

0

0

0

0

0

0

0

0

0

0

0

0

1

1

1

0

0

1

1

0

0

0

0

0

0

0

0

0

0

0

0

0

0

0

0

0

0

0

0

0

0

0

0

0

0

0

600

0

0

0

0

0

0

0

0

0

0

0

0

0

0

0

0

0

0

0

0

0

0

0

0

0

0

0

0

0

0

0

0

0

0

0

0

0

0

0

0

0

0

0

0

4

4

4

4

4

0

0

0

0

0

0

0

0

0

0

0

0

0

0

0

0

0

0

0

596

596

0

0

0

0

0

0

224

224

224

0

0

0

0

0

0

0

0

371

48

48

0

0

0

32

32

0

0

233

233

58

58

0

0

0

1

1

1

0

0

0

0

0

0

0

0

0

0

0

0

0

0

0

657

657

657

657

0

0

598

217

281

100

59

59

0

0

0

0

0

0

0

0

0

0

0

0

0

0

0

0

0

0

0

0

0

0

0

0

0

0

0

0

0

0

0

0

0

0

0

0

0

0

0

0

0

0

0

0

0

0

0

0

0

0

0

0

1

1

1

0

0

0

0

0

1

0

0

0

1

0

0

0

0

0

0

1

0

0

0

0

0

0

0

0

21249

10540

0

0

0

0

0

0

0

0

0

0

0

0

0

0

156

156

140

140

16

16

591

0

0

0

0

0

0

591

0

0

591

591

32

32

32

0

32

0

2641

2269

3

3

0

0

485

457

2

26

0

71

10

61

0

0

0

129

129

0

0

0

0

3

2

1

0

0

0

0

7

7

0

0

14

14

3

3

0

0

5

0

0

5

0

0

5

0

0

0

0

4

0

0

0

0

0

0

0

0

0

1

0

0

0

0

0

18

18

64

64

30

0

30

0

0

40

0

33

7

8

0

1

0

2

0

5

20

0

0

0

9

11

0

0

0

0

9

9

26

0

0

0

26

0

53

53

43

43

55

55

64

62

2

0

0

0

0

0

92

92

5

5

0

0

75

0

12

63

0

0

0

0

0

61

61

1

1

0

0

0

0

0

0

0

0

112

6

106

2

2

0

400

0

239

0

55

2

76

1

2

0

0

0

25

366

232

134

0

0

0

372

372

0

372

6685

1552

164

164

186

0

12

174

1

0

1

27

27

524

454

70

490

490

160

154

0

0

6

0

0

0

0

0

0

3830

1

1

0

0

2107

2107

64

64

1635

1635

0

0

23

22

1

1303

0

0

0

0

0

0

0

0

0

1303

1303

0

0

5

5

0

0

0

0

5

3

0

0

0

0

0

2

0

0

7

0

0

0

5

5

5

0

0

0

1

1

1

1

1

1

0

0

0

0

0

0

0

0

0

379

318

0

0

0

1

1

0

0

0

0

0

0

0

0

0

0

0

0

0

0

0

0

0

0

0

0

0

0

0

190

0

0

0

188

2

9

1

6

1

1

0

0

0

0

0

0

0

0

108

108

8

1

7

2

0

0

0

2

48

48

48

0

0

0

7

0

0

0

7

7

0

0

0

0

0

0

0

0

0

0

0

0

0

0

0

0

6

0

0

0

0

0

0

0

1

1

0

0

1

1

0

0

0

0

0

0

0

0

0

0

4

4

0

0

0

0

0

0

0

0

0

44

0

0

0

0

0

0

18

18

18

26

26

26

0

0

0

6548

0

0

0

0

29

6

0

0

0

0

0

0

1

0

0

0

1

0

0

0

0

4

4

0

0

1

1

0

0

23

0

0

0

0

0

0

0

23

0

0

0

0

0

2

0

0

0

1

0

1

0

0

0

1

18

0

0

0

0

0

0

0

0

0

0

0

0

0

1402

771

0

0

0

0

44

44

2

1

0

0

1

0

0

723

723

0

0

0

0

0

2

2

631

3

3

85

85

0

0

0

0

0

0

32

32

0

0

0

221

221

1

1

18

0

18

1

1

2

2

0

0

0

0

0

0

188

188

80

80

0

0

0

0

191

4

0

0

0

0

4

3

1

187

187

187

0

0

0

0

0

0

0

10

10

0

0

0

0

10

1

0

0

0

1

0

0

0

0

0

0

0

0

8

0

0

0

0

0

0

0

0

0

0

0

0

0

0

0

0

0

0

0

0

6

1

1

1

0

0

0

5

5

5

0

0

0

1

1

1

1

0

4613

1

1

1

0

14

0

0

0

0

14

14

0

0

0

0

3352

3346

0

0

17

0

1210

0

110

8

1993

0

6

2

0

6

6

0

3

3

1

2

1242

2

2

1

1

5

4

0

1

9

0

6

3

208

0

170

4

0

0

34

11

11

586

19

0

562

5

0

0

7

7

405

382

0

23

2

0

2

3

3

3

1

2

0

0

0

1

0

0

0

0

1

1

282

282

282

0

50

54

0

178

0

14

14

0

0

0

0

14

14

0

0

0

0

0

0

0

0

0

0

0

0

0

0

0

4161

0

0

0

0

0

0

4161

0

0

0

1528

0

0

0

0

1

1

701

0

701

826

166

103

69

25

1

162

31

34

235

2632

118

93

25

1019

1019

1428

1

497

775

155

67

62

5

1

1

1

0

0

0

0

0

8

0

0

0

0

0

0

0

0

0

0

0

0

0

0

0

0

0

0

0

0

0

0

0

0

0

0

0

0

0

0

0

0

0

0

0

0

0

0

0

0

8

0

0

0

0

8

8

0

0

0

0

0

0

8

8

0

0

0

0

0

0

0

0

0

0

0

0

0

0

0

0

0

0

0

0

0

0

0

0

0

0

0

0

0

0

17

17

17

17

17

0

17

0

0

0

0

0

0

0

0

0

0

0

0

0

0

0

0

0

0

0

0

0

0

0

0

0

0

0

0

0

0

0

0

0

0

2

2

2

2

2

2

2

5

1

1

1

0

0

0

1

1

1

0

0

0

0

0

0

0

0

3

3

3

3

3

3

1

1

1

1

1

1
